# Smoking, urinary cotinine levels and incidence of visual impairment

**DOI:** 10.1038/s41598-020-79865-z

**Published:** 2021-01-11

**Authors:** So Young Han, Yoosoo Chang, Hocheol Shin, Chul Young Choi, Seungho Ryu

**Affiliations:** 1 Department of Ophthalmology, Kangbuk Samsung Hospital, Sungkyunkwan University School of Medicine, 29 Saemunan-ro, Jongno-gu, Seoul, 03181 Republic of Korea; 2 Center for Cohort Studies, Total Healthcare Center, Kangbuk Samsung Hospital, Sungkyunkwan University School of Medicine, Seoul, Republic of Korea; 3 Department of Occupational and Environmental Medicine, Kangbuk Samsung Hospital, Sungkyunkwan University School of Medicine, Samsung Main Building B2, 250, Taepyung-ro 2ga, Jung-gu, Seoul, 04514 Republic of Korea; 4 Department of Clinical Research Design and Evaluation, SAIHST, Sungkyunkwan University, Seoul, Republic of Korea; 5 Department of Family Medicine, Kangbuk Samsung Hospital, Sungkyunkwan University School of Medicine, Seoul, Republic of Korea

**Keywords:** Diseases, Health care, Risk factors

## Abstract

The longitudinal relationship between smoking status and risk of developing visual impairment (VI) remains unclear. We examined the relationship of smoking status and urinary cotinine level, an objective measure of smoking, with incidence of VI. This cohort study included 279,069 individuals free of VI who were followed for up to 8.8 years (median 4.8 years). VI was defined as when bilateral visual acuity was worse than 0.5 (cutoffs of 0.3 Logarithm of the Minimum Angle of Resolution). During 1,324,429.8 person-years of follow-up, 7852 participants developed new-onset bilateral VI. Self-reported current smoking status was associated with increased risk of developing VI in both men and women, with a stronger association in women (P for interaction = 0.01). Multivariable adjusted hazard ratios (95% confidence intervals) for incident VI comparing current smokers to never-smokers were 1.14 (1.04–1.25) in men and 1.52 (1.28–1.80) in women. Urinary cotinine levels of ≥ 100 ng/ml were significantly associated with increased risk of incident VI, and these associations remained when introducing changes in urinary cotinine and other confounders during follow-up as time-varying covariates. Cigarette smoking assessed based on self-report and urinary cotinine level was associated with increased incidence of VI. Our findings identify smoking as an independent risk factor for VI.

## Introduction

Visual impairment (VI) or blindness is an important public health issue that was estimated to affect 440 million people in 2015 worldwide^1,2^. VI reduces vision-related quality of life, which results in increases of risk of dependency, accidents, falls, and depression^3–6^. VI itself is reported to be related to high risk of mortality^7–9^. Age-related macular degeneration (AMD) and cataracts are the leading ocular diseases related to severe bilateral VI, and the prevalence of these diseases increases with age^10^. However, the cause and mechanism of VI is not fully elucidated, other than being related to specific eye diseases such as AMD, cataract, glaucoma, and diabetic retinopathy. Indeed, Visual acuity (VA) reflects the integrity of the visual system and is considered as a “vital sign” of ocular function^11^. In this context, VI appears to be an important sign of integral ocular health. Therefore, it is important to identify modifiable risk factors for VI to establish preventive strategies to decrease VI-associated health burden and social cost, and preserve quality of life.

Cigarette smoking is the leading preventable cause of premature mortality worldwide and is closely associated with a wide range of diseases^12^. Smoking is associated with worsening of some ocular morbidities, especially AMD and cataracts^13^. Most studies have focused on the detrimental effects of cigarette smoking on individual specific eye diseases such as AMD^14–16^ and cataracts^17,18^. Only a few studies have addressed the relationship between smoking and VI^19–22^, and in existing studies, cigarette smoking was only one of many behavioral factors rather than a main exposure variable, and the results did not provide detailed information about smoking. Furthermore, many previous studies were limited by cross-sectional design, lack of consideration of sex differences, ambiguous temporal relationship of smoking with VI, small sample size, and subjective methods to measure smoking, mainly relying on self-reports. Cotinine, the main metabolite of nicotine, is a reliable objective measure of cigarette smoking status that is useful to minimize the misclassification of smoking status based on self-report^23^.

Despite the health risks related to cigarette smoking and the tobacco control policies in Korea, such as inclusion of health warning labels and images on cigarette packages, increasing smoke-free public places, anti-smoking campaigns and education, and increasing tobacco prices prices^24,25^, the high prevalence of smoking is still observed in Korea, approximately 49.8% of men and 4.2% of women in 2015 according to the 2017 report of the World Health Organization^26,27^.

Therefore, we examined the longitudinal relationships between smoking status, pack-years, and urinary cotinine level as an objective measure of smoking with the risk of developing VI in a large cohort of participants who underwent health screening examinations, while accounting for time-dependent measures of change in smoking status and other covariates during follow-up.

## Results

At baseline, the mean (standard deviation) ages of men and women participants were 38.5 (7.9) years, and 36.6 (7.8) years, respectively (Tables 1, 2). The prevalence of never, former, and current smokers based on self-reports were 27.7%, 35.9%, and 36.4%, respectively, among men and 90.3%, 7.4%, and 2.3%, respectively, among women. Among men, compared with never-smokers, current smokers were more likely to be older, drink alcohol, and have worse lipid profiles and to have high levels of body mass index (BMI), liver enzymes, Homeostatic Model Assessment for Insulin Resistance (HOMA-IR), and total energy intake, while they were less likely to have a high educational level (Table 1). Among women, current smokers were more likely to drink alcohol and have high levels of BMI, triglycerides, but have lower HOMA-IR scores, and they were less likely to have a high educational level than never-smokers (Table 2).

**Table 1:** Baseline characteristics of study participants according to smoking status among men.

Characteristics	Overall	Smoking status			*P* for trend
		Never smoker	Former smoker	Current smoker	
Number	161,808	44,934	58,003	58,871	
Age (years)^a^	38.5 (7.9)	35.7 (7.3)	40.3 (8.5)	38.9 (7.0)	< 0.001
BMI (kg/m^2^)	24.6 (3.0)	24.4 (3.1)	24.6 (2.9)	24.8 (3.1)	< 0.001
Obesity (%)	40.7	37.0	40.8	43.4	< 0.001
Alcohol intake (%)^c^	35.7	22.0	34.3	47.5	< 0.001
Physically active (%)^d^	17.4	17.0	19.5	15.6	< 0.001
High education level (%)^e^	89.0	93.0	88.4	86.3	< 0.001
Hypertension (%)	14.4	11.6	16.7	14.3	< 0.001
Diabetes (%)	4.6	2.6	5.3	5.4	< 0.001
Medication for dyslipidemia (%)	2.5	1.5	3.4	2.3	< 0.001
History of CVD (%)	1.1	0.6	1.5	1.0	< 0.001
Systolic BP (mmHg)^a^	114.8 (11.4)	114.4 (11.1)	115.4 (11.5)	114.6 (11.6)	0.683
Diastolic BP (mmHg)^a^	73.7 (9.3)	72.8 (9.1)	74.4 (9.3)	73.8 (9.5)	< 0.001
Glucose (mg/dl)^a^	97.4 (15.4)	95.2 (13.0)	98.1 (15.1)	98.3 (17.1)	< 0.001
Total cholesterol (mg/dl)^a^	198.9 (34.4)	195.4 (33.3)	200.2 (34.7)	200.4 (34.9)	< 0.001
LDL-C (mg/dl)^a^	128.4 (31.6)	127.0 (31.0)	129.3 (31.7)	128.6 (32.0)	< 0.001
HDL-C (mg/dl)^a^	53.2 (13.0)	54.5 (13.0)	53.8 (13.0)	51.6 (12.8)	< 0.001
Triglycerides (mg/dl)^b^	112 (80–163)	97 (70–139)	111 (79–159)	128 (89–184)	< 0.001
ALT (U/l)^b^	24 (17–35)	23 (17–34)	24 (17–35)	25 (18–36)	< 0.001
GGT (U/l)^b^	30 (20–48)	25 (18–38)	30 (20–47)	35 (23–58)	< 0.001
HOMA-IR^b^	1.31 (0.86–1.97)	1.31 (0.87–1.95)	1.31 (0.86–1.97)	1.32 (0.86–1.99)	0.135
hsCRP (mg/l)^b^	0.5 (0.3–1.0)	0.5 (0.3–1.0)	0.5 (0.3–1.0)	0.6 (0.3–1.1)	< 0.001
Total energy intake^b,^^f^	1631.6 (1291.1–2035.5)	1588.6 (1241.5–2001.6)	1640.9 (1310.4–2029.8)	1655.9 (1311.7–2068.3)	< 0.001
Cotinine level^b,^^g^	34 (34–439)	34 (34–34)	34 (34–34)	830 (292–1457)	< 0.001

Data are presented as ^a^means (standard deviation), ^b^medians (interquartile range), or percentages.

*BMI* body mass index, *BP* blood pressure, *LDL-C* low-density lipoprotein-cholesterol, *HEPA* health-enhancing physically active, *HDL-C* high-density lipoprotein-cholesterol, *HOMA-IR* homeostasis model assessment of insulin resistance, *hsCRP* high sensitivity C-reactive protein.

^c^≥ 20 g of ethanol per day; ^d^≥ health enhancing physically active; ^e^≥ College graduate.

^f^Among 113,085 men with plausible estimated energy intake levels (within three standard deviations from the log-transformed mean energy intake).

^g^Among 115,228 men with available cotinine level.

**Table 2:** Baseline characteristics of study participants according to smoking status among women.

Characteristics	Overall	Smoking status			*P* for trend
		Never smoker	Former smoker	Current smoker	
Number	117,261	105,914	8691	2656	
Age (years)^a^	36.6 (7.8)	36.6 (7.8)	36.8 (7.1)	36.2 (7.5)	0.350
BMI (kg/m^2^)	21.6 (3.1)	21.6 (3.0)	21.8 (3.2)	21.9 (3.4)	< 0.001
Obesity (%)	12.0	11.8	13.7	15.8	< 0.001
Alcohol intake (%)^c^	6.8	5.7	13.3	28.6	< 0.001
Physically active (%)^d^	13.0	12.7	14.6	15.4	< 0.001
High education level (%)^e^	79.1	79.7	75.4	66.9	< 0.001
Hypertension (%)	3.8	3.8	4.0	3.8	0.399
Diabetes (%)	1.5	1.5	1.6	1.8	0.077
Medication for dyslipidemia (%)	1.2	1.2	0.8	0.9	0.002
History of CVD (%)	0.5	0.5	0.8	1.1	< 0.001
Systolic BP (mmHg)^a^	101.8 (11.0)	101.7 (11.0)	102.3 (11.4)	101.8 (10.5)	0.002
Diastolic BP (mmHg)^a^	65.0 (8.2)	65.0 (8.2)	65.3 (8.4)	65.2 (8.2)	0.001
Glucose (mg/dl)^a^	91.0 (10.6)	91.0 (10.5)	91.4 (10.4)	92.0 (13.2)	< 0.001
Total cholesterol (mg/dl)^a^	185.7 (32.0)	185.7 (31.9)	186.3 (32.1)	186.0 (32.1)	0.135
LDL-C (mg/dl)^a^	109.3 (29.1)	109.5 (29.1)	108.2 (29.3)	107.2 (30.0)	< 0.001
HDL-C (mg/dl)^a^	66.4 (15.1)	66.3 (15.1)	66.6 (15.2)	66.8 (16.5)	0.029
Triglycerides (mg/dl)^b^	69 (54–94)	69 (53–93)	70 (54–95)	77 (58–107)	< 0.001
ALT (U/l)^b^	13 (10–17)	13 (10–17)	13 (10–17)	13 (10–18)	< 0.001
GGT (U/l)^b^	13 (10–17)	13 (10–17)	13 (11–18)	15 (12–22)	< 0.001
HOMA-IR^b^	1.10 (0.74–1.61)	1.11 (0.74–1.61)	1.05 (0.70–1.55)	1.06 (0.71–1.62)	< 0.001
hsCRP (mg/l)^b^	0.3 (0.2–0.7)	0.3 (0.2–0.7)	0.3 (0.2–0.7)	0.3 (0.2–0.6)	0.480
Total energy intake^b,f^	1349.1 (990.3–1739.1)	1345.1 (985.5–1731.4)	1422.8 (1065.4–1838.6)	1285.4 (936.2–1674.2)	< 0.001
Cotinine level^b,g^	34 (34–34)	34 (34–34)	34 (34–34)	524 (116–1086)	< 0.001

Data are presented as ^a^means (standard deviation), ^b^medians (interquartile range), or percentages.

*BMI *body mass index, *BP* blood pressure, *LDL-C* low-density lipoprotein-cholesterol, *HEPA* health-enhancing physically active, *HDL-C* high-density lipoprotein-cholesterol, *HOMA-IR* homeostasis model assessment of insulin resistance, *hsCRP* high sensitivity C-reactive protein.

^c^≥ 20 g of ethanol per day; ^d^≥ health enhancing physically active; ^e^≥ College graduate.

^f^Among 84,711 women with plausible estimated energy intake levels (within three standard deviations from the log-transformed mean energy intake).

^g^Among 83,054 women with available cotinine level.

Table 3 shows the relationship between self-reported smoking status, smoking pack-years, and the incidence of binocular VI in men and women separately. During 1,324,429.8 person-years of follow-up (median follow-up of 4.8 years; interquartile range, 2.6–6.9 years; maximum 8.8 years), 7852 participants (men, 3549; women, 4303) developed new-onset binocular VI. Based on self-reports, current smoking was significantly associated with an increased incidence of bilateral VI, and this association was stronger in women (P for interaction = 0.01). After adjustments for potential confounders, multivariable adjusted hazard ratio (HR) and 95% confidence interval (CI) for incident VI comparing former and current smokers to never-smokers were 1.00 (0.92–1.10) and 1.14 (1.04–1.25), respectively, in men and 1.11 (1.00–1.23) and 1.52 (1.28–1.80), respectively, in women. In time-dependent models in which updated smoking status and confounders were treated as time-varying covariates, these associations were also observed. Increasing baseline smoking pack-years were positively associated with the incidence of bilateral VI in a dose–response manner in both men and women (P for trend < 0.05), and this association did not differ by sex (P for interaction = 0.098). For men, multivariable adjusted HR (95% CI) for incident VI comparing < 10, 10–19.9, and ≥ 20 pack-years to 0 pack-year were 0.98 (0.90–1.07), 1.11 (1.01–1.22) and 1.13 (1.01–1.23), respectively (P for trend = 0.008). For women, multivariable adjusted HR (95% CI) for incident VI comparing < 10, and ≥ 10 pack-years to 0 pack-years were 1.20 (1.05–1.37), and 1.28 (0.88–1.87), respectively (P for trend = 0.004). When introducing changes in smoking pack-years and confounders during follow-up as time-varying covariates, the association between pack-years and incident VI was similar in women but attenuated in men.

**Table 3:** Development of visual impairment according to smoking status and smoking pack-years.

	Person-years	Incident cases	Incidence density (per 10^n^ person-years)	Age adjusted HR (95% CI)	Multivariable-adjusted HR^a^ (95% CI)	HR (95% CI)^b^ in the model using time-dependent variables
**Men (n = 161,808)**						
Smoking status						
Never smoker	200,138.0	722	3.6	1.00 (reference)	1.00 (reference)	1.00 (reference)
Former smoker	295,919.1	1379	4.7	1.02 (0.93–1.12)	1.00 (0.92–1.10)	0.95 (0.87–1.04)
Current smoker	297,850.6	1448	4.9	1.17 (1.07–1.28)	1.14 (1.04–1.25)	1.17 (1.07–1.29)
*p for trend*				< 0.001	0.001	< 0.001
Pack-years						
0	265,556.6	1014	3.8	1.00 (reference)	1.00 (reference)	1.00 (reference)
< 10	262,965.8	958	3.6	0.98 (0.90–1.07)	0.98 (0.90–1.07)	0.98 (0.89–1.07)
10–19.9	162,955.8	861	5.3	1.12 (1.02–1.23)	1.11 (1.01–1.22)	1.06 (0.97–1.16)
≥ 20	80,334.6	603	7.5	1.15 (1.03–1.28)	1.13 (1.01–1.23)	1.05 (0.94–1.17)
*p for trend*				0.002	0.008	0.214
**Women (n = 117,261)**						
Smoking status						
Never smoker	473,805.3	3767	8.0	1.00 (reference)	1.00 (reference)	1.00 (reference)
Former smoker	45,004.0	396	8.8	1.11 (1.01–1.24)	1.11 (1.00–1.23)	1.09 (0.98–1.21)
Current smoker	11,712.9	140	12.0	1.57 (1.32–1.85)	1.52 (1.28–1.80)	1.55 (1.30–1.85)
*p for trend*				< 0.001	< 0.001	< 0.001
Pack-years						
0	494,008.5	3973	8.0	1.00 (reference)	1.00 (reference)	1.00 (reference)
< 10	26,362.1	233	8.8	1.23 (1.08–1.41)	1.20 (1.05–1.37)	1.17 (1.02–1.35)
≥ 10	2031.1	28	13.8	1.34 (0.92–1.95)	1.28 (0.88–1.87)	1.29 (0.90–1.85)
*p for trend*				0.001	0.004	0.011

The P value for the interaction of sex and smoking status for risk of bilateral visual impairment was 0.010 (multivariable model).

The P value for the interaction of sex and pack-years for risk of bilateral visual impairment was 0.098 (multivariable model).

*BMI* body mass index, *CI* confidence intervals, *CVD* cardiovascular disease, *HR* hazards ratio.

^a^Estimated from parametric proportional hazard models. Multivariable model was adjusted for age, sex (only for total), center, year of screening exam, BMI, physical activity, alcohol intake, total energy intake, educational level, medication for dyslipidemia, history of CVD, history of diabetes and history of hypertension.

^b^Estimated from parametric proportional hazard models with smoking status, pack-years category, physical activity, alcohol intake, total energy intake, BMI, medication for dyslipidemia, history of diabetes, history of hypertension, and history of CVD as time-dependent categorical variables and baseline age, sex, center, year of screening exam, and education.

Urinary cotinine level, an objective marker of smoking, was associated with increased risk of incident VI in men and women (all P for trend < 0.05), with no significant interaction by sex (P for interaction = 0.742) (Table 4). Multivariable-adjusted HR (95% CI) for incident VI comparing urinary cotinine ≥ 100 ng/ml to < 50 ng/ml was 1.17 (1.08–1.27) in men and 1.22 (1.002–1.48) in women. In the time-dependent model, the association between urinary cotinine levels ≥ 100 ng/ml and incident VI was stronger than in the previous model.

**Table 4:** Development of bilateral visual impairment according to cotinine level.

Cotinine level	Person-years	Incident cases	Incidence density (per 1,000 person-years)	Age sex adjusted HR (95% CI)	Multivariable-adjusted HR^a^ (95% CI)	HR (95% CI)^b^ in the model using time-dependent variables
**Total (n = 198,282)**						
< 50	757,397.9	4043	5.3	1.00 (reference)	1.00 (reference)	1.00 (reference)
50–99	10,199.4	44	4.3	1.05 (0.78–1.42)	1.04 (0.77–1.40)	1.29 (0.95–1.74)
≥ 100	216,012.5	1039	4.8	1.22 (1.13–1.31)	1.18 (1.09–1.27)	1.29 (1.19–1.40)
*P for trend*				< 0.001	< 0.001	< 0.001
**Men (n = 115,228)**						
< 50	381,928.1	1386	3.6	1.00 (reference)	1.00 (reference)	1.00 (reference)
50–99	8620.6	31	3.6	0.99 (0.69–1.41)	0.98 (0.68–1.40)	1.27 (0.90–1.80)
≥ 100	204,317.1	934	4.6	1.20 (1.11–1.31)	1.17 (1.08–1.27)	1.30 (1.19–1.42)
*P for trend*				< 0.001	< 0.001	< 0.001
**Women (n = 83,054)**						
< 50	375,469.8	2657	7.1	1.00 (reference)	1.00 (reference)	1.00 (reference)
50–99	1578.8	13	8.2	1.23 (0.72–2.13)	1.23 (0.71–2.12)	1.34 (0.72–2.49)
≥ 100	11,695.4	105	9.0	1.27 (1.04–1.54)	1.22 (1.002–1.48)	1.25 (1.02–1.53)
*P for trend*				0.014	0.038	0.022

The P value for the interaction of sex and cotinine levels for risk of bilateral visual impairment was 0.742.

*BMI *body mass index, *CI* confidence intervals, *CVD* cardiovascular disease, *HR* hazards ratio.

^a^Estimated from parametric proportional hazard models. Multivariable model was adjusted for age, sex (only for total), center, year of screening exam, BMI, physical activity, alcohol intake, total energy intake, educational level, medication for dyslipidemia, history of CVD, history of diabetes, and history of hypertension.

^b^Estimated from parametric proportional hazard models with cotinine levels category, physical activity, alcohol intake, total energy intake, BMI, medication for dyslipidemia, history of diabetes, history of hypertension, and history of CVD as time-dependent categorical variables and baseline age, sex, center, year of screening exam, and education level as time-fixed variables.

In subgroup analyses (Supplementary Table S1), the association between smoking status and VI was consistently observed without significant interaction by age (< 50 vs. ≥ on years), alcohol intake (< 20 vs. ≥ 20 g/day), health-enhancing physical activity (no vs. yes), BMI (< 25 vs. 25 kg/m^2^), diabetes (no vs. yes), or hypertension (no vs. yes).

In sensitivity analyses using unilateral VI (either right or left VI) as an endpoint instead of bilateral VI, the association between smoking and VI was consistently observed (Supplementary Table S2).

## Discussion

This large cohort study of young and middle-aged Korean men and women demonstrated that current smokers were at higher risk of incident VI than never smokers, and this association remained after adjustments for various confounding factors. These independent and positive associations were stronger in women. Smoking pack-years also showed a dose–response relationship with new-onset VI. In women, the risk increased even with relatively low pack-years (< 10 pack-years) compared to men. The lack of a significant positive association between highest pack-years (≥ 10 pack-years) with incidence of VI in women was probably due to insufficient sample size. Urinary cotinine level, an objective marker of smoking, was associated with increased risk of VI in both men and women, and these associations became evident when changes in urinary cotinine level and other confounders during follow-up were treated as time-varying covariates. Our findings suggest that cigarette smoking is an independent risk factor for VI and that women are more susceptible to the deleterious effects of smoking on vision than men.

A few studies evaluated the effects of smoking on VI. According to the Beaver Dam Eye Study, a prospective cohort study of 9548 individuals aged 43 years or older free of VI at baseline with a maximum follow-up of 20 years, current and former smoking were related to decreased vision, and incident VI was higher in current smokers compared to never-smokers with fully-adjusted odds ratios of 2.01 (95% CI 0.87–4.61)^19^. In a cross-sectional study of 12,233 Chinese participants, presenting VI was associated with smoking (odds ratio: 1.55; 95% CI 1.31–1.83), but this study did not include detailed information on smoking such as current vs. former smoking or smoking pack-years^21^. A cross-sectional study by Merle et al.^22^ using nationally representative French sample demonstrated that heavy smokers with ≥ 20 pack-years had a 1.48 (95% CI 1.03–2.14)-fold increase in the odds of presenting VI compared to never smokers, whereas no association with VI was observed among smokers with < 20 pack-years with a corresponding odds ratio of 0.98 (95% CI 0.72–1.32). Another cross-sectional study by Aljied et al.^20^ based on the Canadian Longitudinal Study on Aging included 30,097 adults aged between 45 and 85 years and showed that the odds (95% CI) of presenting VI in former and current smokers were 1.04 (0.93–1.17) and 1.52 (95% CI 1.25–1.85) compared to never smokers. However, previous studies were limited by cross-sectional design^20–22^, and lack of detailed information regarding smoking such as current vs. former smoking^21^ and smoking pack-years^20,21^. Additionally, all of these studies relied on self-reported smoking and did not incorporate changes in smoking status during follow-up. The present cohort study of relatively young men and women demonstrated positive and independent associations of both subjective and objective measures of cigarette smoking with the development of VI, while incorporating smoking status and confounders at both baseline and follow-up visits.

To date, the pathophysiology of smoking and the incidence of VI remain unclear, however, several explanations to ocular disease or effects on eyes after smoking are suggestive. Cigarette smoke is composed of thousands of hazardous compounds including toxic particles, gases, chemicals, and heavy metals^28,29^. Increased oxidative stress by reactive oxygen species directly and indirectly damage the lens, and heavy metals can accumulate on the lens, which causes cataracts even in young adults^18,29^. Oxidative stress due to free radicals also damages the retina and can cause age-related macular degeneration^13,30^. Smoking damages the optic nerve due to ischemic changes, and furthermore, smoking promotes inflammation that can aggravate the course of uveitis and thyroid-associated orbitopathy^13^. Besides the pre-existing ocular diseases, smoking may cause pathological changes in eyes by decreasing the choroidal vessel circulation^31,32^ and accelerating endothelial apoptosis, which diminishes the endothelial function of the cornea due to reactive oxygen species^29^, and DNA damage^33^. There are some reports of reduced contrast sensitivity associated with low serum levels of manganese and zinc^34^, and activated nicotinic acetylcholine receptors, the functional units of visual processing^35^, in heavy smokers.

In the present study, the associations between current smoking, smoking pack-years, and risk for developing VI were stronger in women, although only a small proportion (2.3%) of women were current smokers. In Korea, the overall prevalence of smoking in 2016 was 39.7% in men and 3.3% in women^36^, similar to our sample. Smoking prevalence remained unchanged for 10 years from 2003 in women, whereas that of men decreased consistently^36^. The mechanism underlying stronger associations of smoking with development of VI among women is not fully understood. Although controversial, there are a few studies suggesting that women are more susceptible to the development of smoking-related diseases than men^37–39^. Additionally, a previous study showed that women are less likely to quit smoking and more likely to transit from being a former smoker to a current smoker^40^. These differences among women might also be attributed to menstrual cycles and hormone variation^41^, greater nicotine dependence^40,42^, or lower level of education^21^. It is also well known that individuals with higher education levels tend to engage in more preventive care and are more likely to have healthy behaviors^21^. In this study, the prevalence of high education attainment was lower among women, which was the lowest in current women smokers. However, after adjustment for education level and other health behaviors, the association between smoking and development of VI remained significant. Further studies are required to confirm these findings and to elucidate the mechanism underlying potential sex differences in the associations between smoking and the risk of VI.

This study’s main strengths lie in its design as a large-scale cohort study of young and middle-aged men and women, and the inclusion of a wide range of confounders as well as detailed information on smoking status based on both subjective and objective measures of smoking status. These factors enabled us to demonstrate independent and dose–response associations of subjective and objective smoking with new-onset of VI and to identify differences between men and women while incorporating changes in smoking status and other covariates during follow-up.

There are several limitations in this study. First, data for best-corrected VA was not available. We defined VI based on presenting VA of participants whose refractive errors may have been partially corrected with their own glasses/lenses. However, presenting VA reflects the real vision of individuals in everyday life^9^ and is widely used in population-based and other clinical studies^8,9,20^. Second, the present study used both self-reported and an objective estimate of smoking status, however, the half-life of nicotine metabolism is about 16–20 h. Therefore, measurement errors may result in errors in estimates of actual smoking status of irregular smokers, or people who prevised the cotinine test and avoided smoking temporarily before health screening examination. Third, the history of ocular diseases was determined based on self-reporting of physician-diagnosed ocular diseases. Specific causes of VI were not addressed in this study. Thus, we cannot exclude some degree of residual confounding related to measurement errors and other unmeasured confounding factors, which might have affected the observed associations between smoking and incident VI. Fourth, environmental tobacco smoking was not considered. In Korea, the prevalence of environmental tobacco smoking in workplace was 57.2% among men and 38.7% among women in 2013^43^. As second-hand smoking also increases incidence of diseases, such as lung cancer and deaths^44^, VI in non-smokers may have affected by environmental tobacco smoking. Fifth, the data on urinary creatinine concentrations were not available, limiting our ability to control for dilution effects^45,46^. Sixth, the exposure to occupational hazards such as organic solvents may affect as potential confounding factors to VI. Organic solvents affect the retina and optic nerve with neurotoxicity resulting color vision loss and lower visual function^47,48^. In our study, participants are mostly white-collar workers; thus the results are less likely to be affected by occupational hazards related to organic solvents. Additionally, nicotine metabolism can vary across ethnicity/race and our findings might not be generalizable to other race/ethnic groups. Finally, our study findings were derived from relatively healthy young and middle-aged educated Koreans with high accessibility to healthcare, and might not be generalizable to other populations with different characteristics.

In the present cohort study, both subjective and objective measures of smoking were positively and independently associated with an increased risk of development of VI in both men and women, with a stronger association in women. Current smoking was consistently associated with increased risk of VI, whereas former smoking was not. Our results suggest that smoking is an independent risk factor of decreased vision and that incidence of VI may be preventable by cessation of smoking or avoidance of smoking initiation. Tobacco control policies regarding health promotion and education would have important role to prevent VI in addition to other smoking-induced diseases. Further studies are needed to evaluate the exact pathogenesis of VI according to smoking and sex differences in biological and physiological effects of smoking on VI.

## Methods

### Study population

The Kangbuk Samsung Health Study is a cohort study of Korean adults who underwent comprehensive annual or biennial health examinations at the clinics of Kangbuk Samsung Hospital Total Healthcare Screening Center in Seoul and Suwon, South Korea^49^. In Korea, all employees are mandatory for health care by law under National Health Insurance. Eye examination with visual acuity is one of the essential items in basic health care in Korea. This study was designed to use data routinely collected as part of health screening examinations where questionnaires, blood tests, imaging examination, and procedures (e.g., ultrasound, endoscopy) are components of health screening exams^49,50^. More than 80% of the participants were employees of companies and local governmental organizations or their spouses. In South Korea, the Industrial Safety and Health Law requires annual or biennial health-screening exams of all employees to be offered free of charge. An office worker receives biennial health-screening examinations, and a blue-collar worker receives annual health-screening examinations. The remaining participants voluntarily paid for screening examinations at the health screening center.

**Figure 1:**
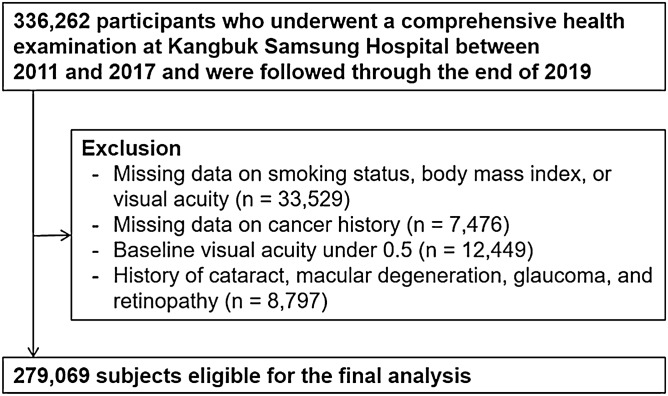
Flow diagram for the selection of the study subjects.

The present analysis included all study participants who received comprehensive health examinations including vision tests between 2011 and 2017, and who underwent at least one follow-up vision test prior to December 31, 2019 (N = 336,262; Fig. 1). A total of 57,193 participants (age range 18–89 years old) met one of the following exclusion criteria at baseline: missing data for smoking status, BMI, or VA (n = 33,529); history of cancer (n = 7476); VI defined as presenting VA < 0.5 (n = 12,449); or history of physician-diagnosed ocular diseases including cataracts, macular degeneration, glaucoma, and retinopathy (n = 8797). Some individuals met more than one exclusion criterion, and thus a total of 279,069 participants were included in the final analysis. The baseline characteristics according to missing visual acuity information is presented as Supplementary Table S3.

We followed all related tenets of the Declaration of Helsinki and this study was approved by the Institutional Review Board of Kangbuk Samsung Hospital (KBSMC 2020-04-025), which waived the requirement for written informed consent due to the use of de-identified data obtained as part of routine health screening examinations.

### Data collection

Physical measurements, vision tests, and laboratory measurements of urine and blood were assessed every 1–2 years as part of the routine screening program at baseline and follow-up visits. Demographic characteristics, smoking habits and other lifestyle factors, and medical history were collected at each visit using standardized, self-administered questionnaires as previously described^49^. Physician-diagnosed eye diseases including cataracts, glaucoma, dry eye, macular degeneration, and any type of retinopathy were also assessed. Questions regarding smoking included lifetime and current smoking status, smoking duration, and number of cigarettes per day. Participants who had smoked < 100 cigarettes during their lifetime were classified as never-smokers. Participants who had smoked > 100 cigarettes in their lifetime were further categorized into two groups as follows: current smokers and former smokers who no longer smoked at all at the time of their screening examination. In Korea, 20 cigarettes are sold per pack. Pack-years were calculated as the product of smoking duration and cigarettes per day divided by 20. Alcohol consumption was categorized as none, < 20 g of ethanol/day, and ≥ 20 g of ethanol/day. The Korean version of the International Physical Activity Questionnaire Short Form was used to assess physical activity level^51,52^ which was classified into three categories: inactive, minimally active, and health-enhancing physically active as described previously^53^. Usual dietary intake over the past year was assessed using a Korean version of a 106-item self-administered food frequency questionnaire^54^. CVD and cancer were defined as physician-diagnosed heart disease or stroke and physician-diagnosed malignancy of any type, respectively.

Height, weight, and blood pressure (BP) were measured by trained nurses. Obesity was defined as BMI ≥ 25 kg/m^2^ according to Asian-specific criteria^55^. Hypertension was defined as systolic BP ≥ 140 mmHg, diastolic BP ≥ 90 mmHg, self-reported history of hypertension, or current use of antihypertensive medications.

Fasting blood measurements included glucose, glycated hemoglobin (HbA1c), lipid profiles, insulin, and high sensitivity C-reactive protein. Insulin resistance was assessed with the following HOMA-IR equation: fasting blood insulin (uU/ml) × fasting blood glucose (mmol/l)/22.5. Diabetes mellitus (DM) was defined as fasting serum glucose ≥ 126 mg/dl, HbA1c ≥ 6.5%, or current use of antidiabetic medications.

Urinary cotinine level was measured using the DRI Cotinine Assay (Microgenics Corp., Fremont, CA, USA) with a modular P800 chemistry analyzer (Roche Diagnostics, Tokyo, Japan). A urine cotinine cut-off point of 100 ng/ml is used as a reference point in the screening center of our hospital, since nonsmoker urine levels are reported not to exceed 100 ng/ml^56^. Urinary cotinine 50 ng/ml has also been widely used to distinguish tobacco use vs. no tobacco use according to the SRNT Subcommittee on Biochemical Verification^57^. Therefore, in the present study, urinary cotinine levels were categorized into three groups: (1) < 50 ng/ml; (2) 50–99 ng/ml; (3) ≥ 100 ng/ml.

Presenting VA with habitual correction if worn was assessed for each eye using an international standard chart that is based on Early Treatment Diabetic Retinopathy Study (ETDRS) scales at 3 m with distance. Presenting VA (either VA of naked eyes or corrected VA) was used because it represents the VA of individuals in the real world^9^. VA was recorded as a decimal, which is the usual unit in clinical practice in Korea, and then converted to Logarithm of the Minimum Angle of Resolution (logMAR) scale. For the main analysis, VI was defined as when VA was worse than 0.5 (cutoffs of 0.5; 20/40 Snellen; 0.3 logMAR) in both eyes^19,20,22^.

### Statistical analysis

The baseline characteristics of the study participants are presented according to smoking status. The relationship between smoking status and incident VI differed significantly by sex; therefore, analyses were performed separately for men and women. Pack-years were categorized as never (0), > 0–10, > 10–20, and ≥ 20 pack-years. Few women participants (n = 64) were identified as having ≥ 20 pack-years and were thus combined with the category of > 10–20 years, resulting in a category of ≥ at pack-years in women.

The primary endpoint was the development of binocular VI, defined as VA < 0.5 in both eyes. The participants were followed from their baseline examination until either the development of binocular VI or the last health exam conducted prior to December 31, 2019, whichever came first. The incidence rate was calculated as incident cases divided by the number of person-years of follow-up. Since new-onset VI, if it did occur, would have occurred at an unknown time point between the visit at which VI was diagnosed by vision tests and the prior visit, a parametric proportional hazards model was used to account for this type of interval censoring (*stpm* command in Stata)^58^. In these models, the baseline hazard function was parameterized with restricted cubic splines in log time with four degrees of freedom.

The HR and 95% CI were calculated for incident VI according to smoking status, pack-years smoked, and urinary cotinine levels. Models were initially adjusted for age and then further adjusted for BMI, alcohol intake (0 g/day, < 20 g/day, ≥ 20 g/day, or unknown), physical activity level (inactive, minimally active, health-enhancing physically active, or unknown), education level (high school graduate or less, community college or university graduate, graduate school or more, or unknown), total calorie intake (in quintiles or missing), history of diabetes (no vs. yes), history of hypertension (no vs. yes) and history of cardiovascular disease (no vs. yes) (multivariable adjusted model). To evaluate the effects of changes in smoking status and other covariates over time during follow-up, we conducted additional analyses introducing smoking status and other covariates as time-varying covariates. The proportional hazards assumption was assessed by examining graphs of estimated log (− log) survival; ultimately, no violations of the assumption were found. To test for linear trends, we included the median value of each category (pack-years and urinary cotinine) as continuous variables in the models.

Subgroup analyses were performed by age (< 50 vs. ≥ 50 years), alcohol intake (< 20 vs. ≥ 20 g/day), health-enhancing physical activity (no vs. yes), BMI (< 25 vs. 25 kg/m^2^), diabetes (no vs. yes), and hypertension (no vs. yes). Interactions between smoking category and subgroup characteristics were tested using likelihood ratio tests that compared models with vs. without multiplicative interaction terms.

All analyses were carried out using STATA version 16.0 (Stata Corp LP, College Station, TX, USA). All P values less than 0.05 were considered to be statistically significant.

## Supplementary Information

Supplementary Information

## Data Availability

All data generated or analyzed during this study are included in this published article.
