# Supplementary material for: Smoking, urinary cotinine levels and incidence of visual impairment
Source: Sci Rep. 2021 Jan 11;11:398. doi: 10.1038/s41598-020-79865-z (PMC7801542; doi:10.1038/s41598-020-79865-z)
Supplement: Supplementary file 1 — Supplementary Information [file 41598_2020_79865_MOESM1_ESM.docx]

**Smoking, urinary cotinine levels and incidence of visual impairment**

So Young Han, Yoosoo Chang, Hocheol Shin, Chul Young Choi, Seungho Ryu

**Supplementary Table S1.** Hazard ratios ^a^ (95% CIs) of bilateral visual impairment according to smoking status in clinically relevant subgroups.

| **Subgroup** | **Smoking status** | | | ***p* for trend** | ***p* for interaction** |
| --- | --- | --- | --- | --- | --- |
|  | **Never smoker** | **Former smoker** | **Current smoker** |  |  |
| **Age** |  |  |  |  | 0.135 |
| <50 years (N=257,648) | 1.00 (reference) | 1.11 (1.04-1.19) | 1.30 (1.20-1.40) | <0.001 |  |
| ≥50 years (N=21,421) | 1.00 (reference) | 1.28 (1.10-1.47) | 1.27 (1.06-1.52) | 0.001 |  |
| **Alcohol intake** |  |  |  |  | 0.488 |
| <20 g/day (N=202,455) | 1.00 (reference) | 1.08 (1.00-1.17) | 1.24 (1.13-1.36) | <0.001 |  |
| ≥20 g/day (N=63,996) | 1.00 (reference) | 0.96 (0.84-1.11) | 1.15 (1.01-1.31) | 0.010 |  |
| **HEPA** |  |  |  |  | 0.261 |
| No (N=231,799) | 1.00 (reference) | 1.04 (0.97-1.13) | 1.19 (1.10-1.30) | <0.001 |  |
| Yes (N=42,614) | 1.00 (reference) | 1.13 (0.99-1.30) | 1.34 (1.16-1.56) | <0.001 |  |
| **BMI** |  |  |  |  | 0.621 |
| <25 kg/m^2^ (N=199,124) | 1.00 (reference) | 1.06 (0.98-1.14) | 1.25 (1.14-1.36) | 0.001 |  |
| ≥25 kg/m^2^ (N=79,945) | 1.00 (reference) | 1.09 (0.97-1.22) | 1.20 (1.07-1.35) | 0.002 |  |
| **Diabetes** |  |  |  |  | 0.274 |
| No (N=269,927) | 1.00 (reference) | 1.07 (1.00-1.15) | 1.21 (1.12-1.31) | <0.001 |  |
| Yes (N=9,137) | 1.00 (reference) | 1.06 (0.83-1.35) | 1.43 (1.13-1.81) | 0.003 |  |
| **Hypertension** |  |  |  |  | 0.361 |
| No (N=251,009) | 1.00 (reference) | 1.06 (0.99-1.14) | 1.21 (1.12-1.31) | <0.001 |  |
| Yes (N=27,657) | 1.00 (reference) | 1.15 (0.98-1.35) | 1.37 (1.15-1.62) | <0.001 |  |

^a^ Estimated from parametric proportional hazard models. Multivariable model was adjusted for age, sex (only for total), center, year of screening exam, BMI, physical activity, alcohol intake, total energy intake, educational level, medication for dyslipidemia, history of CVD, history of diabetes, and history of hypertension

Abbreviations: BMI, body mass index; CI, confidence intervals; CVD, cardiovascular disease; HEPA, health-enhancing physically active.

**Supplementary Table S2. Development of unilateral visual impairment ^a^ by smoking status, smoking pack-years and cotinine levels**

| **Category** | **Total** | **Multivariable-adjusted HR^b^ (95% CI)** | | **p for interaction** |
| --- | --- | --- | --- | --- |
|  |  | **Men** | **Women** |  |
| Smoking status |  |  |  | 0.052 |
| Never smoker |  | 1.00 (reference) | 1.00 (reference) |  |
| Former smoker |  | 1.01 (0.96-1.07) | 1.09 (1.01-1.17) |  |
| Current smoker |  | 1.11 (1.05-1.18) | 1.31 (1.15-1.49) |  |
| p for trend |  | <0.001 | <0.001 |  |
| Pack-years |  |  |  | 0.124 |
| 0 |  | 1.00 (reference) | 1.00 (reference) |  |
| <10 |  | 0.97 (0.91-1.02) | 1.07 (0.97-1.18) |  |
| 10-19.9 |  | 1.05 (0.99-1.11) | 1.17 (0.88-1.55)^c^ |  |
| ≥20 |  | 1.09 (1.02-1.17) |  |  |
| p for trend |  | 0.005 | 0.103 |  |
| Cotinine level |  |  |  | 0.168 |
| <50 | 1.00 (reference) | 1.00 (reference) | 1.00 (reference) |  |
| 50-99 | 1.06 (0.88-1.28) | 0.96 (0.77-1.20) | 1.43 (1.01-2.04) |  |
| ≥100 | 1.13 (1.08-1.19) | 1.13 (1.07-1.19) | 1.16 (1.01-1.34) |  |
| p for trend | <0.001 | <0.001 | 0.019 |  |

^a^ Visual impairment was defined as lesser than 0.5

^b^ Estimated from parametric proportional hazard models. Multivariable model was adjusted for age, sex (only for total), center, year of screening exam, BMI, physical activity, alcohol intake, total energy intake, educational level, medication for dyslipidemia, history of CVD, history of diabetes, and history of hypertension

^c^ Data combined with the category of >10-20 pack-years in women

Abbreviations: BMI, body mass index; CI, confidence intervals; CVD, cardiovascular disease; HR, hazards ratio.

**Supplementary Table S3.** Baseline characteristics according to missing information on visual acuity data

| Characteristics | Visual acuity data available | Visual acuity data missing | P value |
| --- | --- | --- | --- |
| Number | 279,069 | 1,143 |  |
| Age (years)^a^ | 37.7 (7.9) | 42.9 (10.3) | <0.001 |
| BMI (kg/m2) | 23.3 (3.4) | 23.1 (3.4) | 0.038 |
| Men (%) | 58.0 | 48.4 | <0.001 |
| Obesity (%) | 28.7 | 38.2 | <0.001 |
| Alcohol intake (%)^c^ | 24.0 | 24.1 | 0.956 |
| Physically active (%)^d^ | 15.5 | 15.6 | 0.971 |
| High education level (%)^e^ | 84.8 | 78.4 | <0.001 |
| Hypertension (%) | 9.9 | 16.8 | <0.001 |
| Diabetes (%) | 3.3 | 7.2 | <0.001 |
| Medication for dyslipidemia (%) | 1.9 | 4.2 | <0.001 |
| History of CVD (%) | 0.9 | 1.2 | 0.180 |
| Systolic BP (mmHg)^a^ | 109.3 (13.0) | 109.6 (13.8) | 0.483 |
| Diastolic BP (mmHg)^a^ | 70.1 (9.9) | 70.6 (10.3) | 0.085 |
| Glucose (mg/dL)^a^ | 94.7 (13.9) | 97.3 (19.3) | <0.001 |
| Total cholesterol (mg/dL)^a^ | 193.4 (34.0) | 192.2 (35.1) | 0.284 |
| LDL-C (mg/dL)^a^ | 120.4 (32.0) | 118.7 (32.1) | 0.095 |
| HDL-C (mg/dL)^a^ | 58.8 (15.4) | 59.0 (15.9) | 0.564 |
| Triglycerides (mg/dL)^b^ | 90 (64-135) | 88 (62-135) | 0.162 |
| ALT (U/L)^b^ | 18 (13-28) | 17 (12-26) | 0.001 |
| GGT (U/L) ^b^ | 20 (13-36) | 19 (13-33) | 0.014 |
| HOMA-IR^b^ | 1.22 (0.80-1.82) | 1.20 (0.78-1.80) | 0.536 |
| hsCRP (mg/L)^b^ | 0.4 (0.2-0.9) | 0.4 (0.2-1.0) | 0.608 |
| Total energy intake^b, f^ | 1516.1 (1150.2-1919.6) | 1531.8 (1164- 1943.9) | 0.308 |
| Cotinine level^b, g^ | 34 (34-34) | 34 (34-34) | 0.338 |

Data are presented as ^a^means (standard deviation), ^b^medians (interquartile range), or percentages.

^c^ ≥ 20 g of ethanol per day; ^d^≥ health enhancing physically active; ^e^≥ College graduate.

^f^ among 198,335 participants with plausible estimated energy intake levels (within three standard deviations from the log-transformed mean energy intake).

^g^ among 198,282 participants with available cotinine level

Abbreviations: BMI, body mass index; BP, blood pressure; LDL-C, low-density lipoprotein-cholesterol; HEPA, health-enhancing physically active; HDL-C, high-density lipoprotein-cholesterol; HOMA-IR, homeostasis model assessment of insulin resistance; hsCRP, high sensitivity C-reactive protein.
